# Factors that influence field hockey footwear selection: An online survey

**DOI:** 10.1002/jfa2.12019

**Published:** 2024-05-29

**Authors:** Christopher R. Derry, Hylton B. Menz, Katrine Okholm Kryger, Athol Thomson, Caoimhe Hoey, Daniel R. Bonanno

**Affiliations:** ^1^ Discipline of Podiatry School of Allied Health Human Services and Sport La Trobe University Melbourne Victoria Australia; ^2^ La Trobe Sport and Exercise Medicine Research Centre School of Allied Health Human Services and Sport La Trobe University Melbourne Victoria Australia; ^3^ Faculty of Sport, Health and Applied Science St Mary's University Twickenham London UK; ^4^ Sports and Exercise Medicine Queen Mary University of London London UK; ^5^ Aspetar Orthopaedic and Sports Medicine Hospital FIFA Medical Centre of Excellence Doha Qatar; ^6^ Bon Secours Hospital Galway Galway Ireland

**Keywords:** female, hockey, male, questionnaire, shoes

## Abstract

**Background:**

Little is known about factors that influence footwear selection by field hockey players.

**Methods:**

An online survey was used to collect data on participant demographics and physical characteristics, factors influencing footwear selection, perceptions regarding footwear design features on injury and performance, and experiences regarding usability. Nominal and ordinal data were described as absolute frequencies and relative frequencies. Free text responses were analysed using content analysis. Sex‐related differences in quantitative and qualitative data were explored.

**Results:**

A total of 401 hockey players completed the survey. Participants reported that fit, comfort, support, and cushioning were the most important factors when selecting hockey footwear. Most hockey players believed that stud design could influence athletic performance (65%) and injury risk (63%) but reported having no preference on outsole design or stud shape. Most participants (63%) used hockey‐specific footwear, but 46% of female hockey players did not, with 40% using trail running footwear instead. Qualitative analysis revealed that hockey players, particularly female participants, encounter difficulties finding properly fitting footwear. They desire more options for wide or narrow feet and face challenges in accessing suitable hockey shoes due to limited choices and availability.

**Conclusions:**

With over a third of field hockey players not using hockey‐specific footwear, future research should attempt to understand the reasons and assess the impact of different footwear features on comfort, performance, injury risk, and usability.

## 1 BACKGROUND

Field hockey is an Olympic sport played worldwide, with 137 national associations from five continents recognised by the International Hockey Federation [1]. Hockey is a physically demanding sport, requiring players to run at various intensities, encompassing accelerations and decelerations and rapid changes of direction [2, 3]. A recent systematic review found that the incidence of injury in hockey ranged between 4.5 and 57.9/1000 h, with injuries most commonly affecting the lower extremities [4].

Given the high demands of the game, optimal field hockey footwear design is crucial for players' performance, comfort and safety. As hockey is typically played on artificial grass pitches, the outsoles of hockey footwear are designed to maximise footwear‐surface traction to augment performance. Other footwear features, such as toe guards and reinforced uppers, are designed to provide safety through protection from ball or stick impacts. While hockey footwear exists, anecdotally, some players wear footwear designed for other sports (e.g., football, trail running, etc.), suggesting unmet player needs.

There is only limited research investigating field hockey footwear. One study on elite male hockey players found that running faster and playing on harder surfaces increased tibial impacts, yet footwear choice had no significant effect on tibial impacts [5]. Surveys of footwear preferences in other sports (e.g., basketball [6], football [7, 8] and tennis [9]) have found that footwear selection is based on multiple individual factors, including playing position, comfort, foot shape and foot volume. At present, it remains unknown what factors influence the selection of hockey footwear, what gaps exist in the footwear marketplace, or what effects footwear selection can have on injury and performance. Given this, it is reasonable to suggest that there is both a lack of understanding, and need to comprehend, hockey players' footwear perceptions, demands and expectations.

This study aimed to identify factors influencing field hockey players' footwear selection, perceptions regarding footwear design features on injury and performance and experiences regarding usability. A secondary aim was to explore sex‐related differences across these domains. In doing so, the findings of this survey will inform hockey footwear research, future footwear design and player education.

## 2 METHODS

This study was an observational cross‐sectional study using an online‐based survey consisting of non‐randomised open‐ and closed‐ended questions. The study was approved by the La Trobe University Human Ethics Committee (HEC21321) and informed consent was obtained from all participants prior to the recruitment. The study was performed in accordance with the World Medical Association's Declaration of Helsinki [10]. The findings from this study are reported in accordance with the Checklist for Reporting Results of Internet E‐Surveys (CHERRIES; Supporting Information S1) [11].

### 2.1 Survey development

The survey was developed using the following steps to ensure survey completeness, usability, relevance and content validity: (i) construct determination: the research team identified core constructs by reviewing relevant literature on both field hockey and athletic footwear. These constructs included participant specifics, perceptions of field hockey footwear, preferences, beliefs, purchasing behaviours and overall satisfaction with available footwear options; (ii) item generation: with the constructs defined, the research team generated survey items to address each of them; (iii) survey design: the research team structured the survey, considering factors such as target audience, mode of administration and clarity and objectivity in question wording; and (iv) item refinement: survey items were reviewed and refined by the research team to ensure alignment with constructs to ensure that the survey answered the research question [12]. The survey was then piloted on five adult field hockey players from a Victorian Premier League hockey squad, including two with international‐level experience and three with national‐level experience. These participants were considered suitable pilot candidates given their expertise in the physical demands of field hockey and their broad experience as hockey footwear users. They were briefed on the survey's purpose and instructed to assess its comprehensiveness, user‐friendliness, relevance and if the survey measured what we intended to measure (i.e., content validity). Following the piloting phase, no amendments were deemed necessary as the participants expressed satisfaction with the survey and did not propose any changes. The survey (Supporting Information S2) consisted of a demographics and physical characteristics section (13 questions), a footwear preferences section (14 questions) and a footwear beliefs and experiences section (three questions). In total, there were 18 multiple‐choice questions, nine open‐ended questions and three sets of Likert questions. When using Likert questions to measure the perceived importance of footwear preferences (questions 20 and 23), responses were scored on a five‐point scale ranging from ‘not important’ to ‘important’. Likewise, when using Likert questions to measure footwear beliefs (question 29), responses were scored on a five‐point scale ranging from ‘strongly disagree’ to ‘strongly agree’. The survey was available online as an open survey (REDcap, Vanderbilt University, Nashville, USA) between October 2021 and February 2023.

### 2.2 Participants

Participants were recruited through advertisement posters, social media (Twitter [now X], Instagram, Facebook and LinkedIn) and email. To be eligible for study inclusion, participants had to be aged 18 years or older, able to read and understand basic English, be a registered field hockey player in an organised competition and play at least one game per week during competitive season. Participation was voluntary and no incentives were offered to participants.

### 2.3 Sample size

An *a priori* sample size calculation (Qualtrics, Provo, USA) estimated that 385 participants were required for the study, based on the following: (i) approximately 30 million people play field hockey worldwide [13], (ii) a confidence level of 95% and (iii) a margin of error of 5%.

### 2.4 Data analysis

All data were cleaned to remove any participants that did not meet the study eligibility criteria. Remaining data were analysed using IBM SPSS Statistics version 28.0 (IBM Corp). Participant physical characteristics were reported as means with standard deviations. Nominal and ordinal data were described as absolute frequencies (*n*) and relative frequencies (%). Free text responses were qualitatively analysed using a conventional content analysis approach, whereby responses were inductively coded, and then categorised into themes [14]. The data were initially coded by one researcher (CRD), with coding second checked by another researcher (DRB). Differences in coding were resolved via discussion. All costs are reported in Australian dollars. At the time of manuscript submission (October 2023), A$1 was worth approximately €0.60, £0.52 and US$0.63 [15].

## 3 RESULTS

### 3.1 Participant profile

A total of 409 surveys were completed, but eight surveys were removed as the responders did not meet the eligibility criteria (all were 17 years of age). The remaining 401 surveys were completed by adult field hockey players (225 female, 175 male, 1 non‐binary; mean [SD] age, 34.6 [12.3] years; height, 172.6 [10.5] cm; mass, 75.4 [15.5] kg; shoe size, 7.9 [2.1] US) from 10 countries (Table 1). Participants mostly competed at a recreational (e.g., club grades, social; *n* = 164, 41%) or first grade (highest grade within immediate region; *n* = 113, 28%) level, were experienced players (years of play, 22.4 [11.2] years), played several times throughout a typical week (3.0 [1.4] sessions per week) and mostly played on a water‐synthetic surface (*n* = 260, 65%; Table 1). The distribution of strikers, midfielders, defenders and goalkeepers that completed the survey was proportionate with the number of players in these positions within each field hockey team (Table 1).

**TABLE 1:** Participant physical characteristics, ethnicity, playing experience, playing level, playing position and playing surface (*n* = 401).

	Female	Male	Non‐binary	Total
	(*n* = 225)	(*n* = 175)	(*n* = 1)	(*n* = 401)
Age (years)	32.8 (12.0)	37.0 (12.4)	35.0 (−)	34.6 (12.3)
Height (cm)	166.2 (8.4)	180.7 (6.5)	175.0	172.6 (10.5)
Mass (kg)	68.4 (13.0)	84.4 (13.8)	80.0	75.4 (15.5)
Shoe size (US)	6.6 (1.6)	9.7 (1.5)	7.0	7.9 (2.1)
Years of play	21.4 (11.0)	23.7 (11.4)	27.0	22.4 (11.2)
Sessions per week	3.0 (1.5)	3.1 (1.3)	3.0	3.0 (1.4)
Playing level (*n*) (rec/fg/nl/int)	100/61/37/27	63/52/37/23	1/0/0/0	164/113/74/50
Position (*n*) (stk/mid/def/gk)	62/78/71/14	41/46/77/11	1/0/0/0	104/124/148/25
Surface (*n*) (wtr/hy/sd/grs)	152/45/22/6	107/47/21/0	1/0/0/0	260/92/43/6
Ethnicity (*n*)				
Oceanian	136	106	0	242
North‐West European	76	57	1	134
South and Eastern European	3	3	0	6
South‐East Asian	5	5	0	10
North‐East Asian	2	2	0	4
Southern and Central Asian	0	1	0	1
Peoples of the Americas	2	0	0	2
Sub‐Saharan African	1	1	0	2

*Note*: Values are *n* (SD) unless otherwise stated.

Abbreviations: def, defender; fg, first grade; gk, goalkeeper; grs, grass; hy, hybrid synthetic; int, international; mid, midfielder; nl, national league; rec, recreational; sd, sand‐based synthetic; stk, striker; wtr, water‐based synthetic.

### 3.2 Footwear use

#### 3.2.1 Footwear style, brands and usage

Most participants used field hockey‐specific footwear (*n* = 249/401, 62%; female = 55%; male = 72%) or trail running footwear (*n* = 137/401, 34%; female = 40%, male = 26%). The remaining participants (*n* = 15/401) used road running footwear (recreational = 7; first‐grade = 1), synthetic/astro turf football footwear (recreational = 2; national league = 2; international = 2) or tennis footwear (recreational = 1) (all <2%). Twenty‐two footwear brands were used, with the three most common accounting for 76% of usage (*n* = 306/401). Discrepancies in brand usage were observed based on sex, ethnicity and geographical region (Table 2).

**TABLE 2:** Footwear brands worn by sex, ethnicity and geographical region (*n* = 401).

	ASICS (*n* = 182)	Adidas (*n* = 69)	Salomon (*n* = 55)	Mizuno (*n* = 25)	Brooks (*n* = 20)	Osaka (*n* = 11)	Nike (*n* = 8)	Grays (*n* = 5)	Hoka (*n* = 5)	Saucony (*n* = 5)	Other (*n* = 15)
Sex											
Female (*n* = 225)	99	34	40	11	17	1	5	3	3	2	9
Male (*n* = 175)	83	35	14	14	3	10	3	2	2	3	6
Non‐binary (*n* = 1)	0	0	1	0	0	0	0	0	0	0	0
Ethnicity											
Oceanian (*n* = 242)	123	20	35	18	13	7	7	3	3	4	8
North‐West European (*n* = 134)	45	41	18	6	7	4	1	2	2	1	7
South and Eastern European (*n* = 6)	2	2	1	1	0	0	0	0	0	0	0
South‐East Asian (*n* = 10)	5	4	1	0	0	0	0	0	0	0	0
North‐East Asian (*n* = 4)	4	0	0	0	0	0	0	0	0	0	0
Southern and Central Asian (*n* = 1)	0	1	0	0	0	0	0	0	0	0	0
Peoples of the Americas (*n* = 2)	1	1	0	0	0	0	0	0	0	0	0
Sub‐Saharan African (*n* = 2)	2	0	0	0	0	0	0	0	0	0	0
Geographical region											
Oceania (*n* = 333)	163	33	50	23	19	10	8	4	5	5	12
Europe (*n* = 60)	16	31	5	2	1	1	0	1	0	0	3
Asia (*n* = 3)	2	1	0	0	0	0	0	0	0	0	0
America (*n* = 3)	0	3	0	0	0	0	0	0	0	0	0
Africa (*n* = 2)	1	1	0	0	0	0	0	0	0	0	0

*Note*: Other = Combined frequencies for Dita, Dunlop Volley, Gryphon, Kookaburra, Jazba, Michelin, New Balance, Nvii, Scott, Sketchers, Spartan and Topo Ultratrail (all >5). Values are *n*.

**FIGURE 1:**
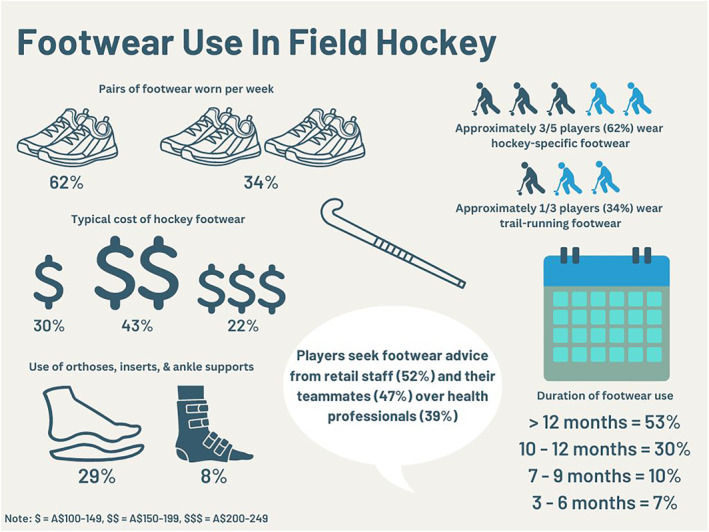
Infographic of footwear use in field hockey.

Most participants used one (*n* = 249/401, 62%; female = 55%, male = 72%) or two pairs of footwear (*n* = 136/401, 34%; female = 40%, male = 26%) in a typical week (Figure 1). Participants using between three to eight pairs per week (*n* = 16/401, 4%) was less common (recreational = 11; first‐grade = 1, national league = 2; international = 2). Most participants used the same footwear irrespective of the playing surface (*n* = 361/401, 90%; female = 91%, male = 89%). The remaining participants (*n* = 40/401, 10%) indicated that they wear different footwear for different surfaces, with free text responses indicating that the main reasons for doing so was improved grip/traction (*n* = 21/40, 53%), different footwear styles used for different surfaces (e.g., trail or hockey footwear depending on the surface) (*n* = 8/40, 20%), footwear durability (*n* = 5/40, 13%), comfort (*n* = 4/40, 10%) and foot support (*n* = 3/40, 8%).

#### 3.2.2 Supplementary shoe inserts and external ankle supports

Approximately one‐third of participants (*n* = 142/401, 35%; female = 39%, male = 31%) used supplementary shoe inserts or external ankle supports when playing field hockey, which mostly consisted of foot orthoses (*n* = 97/401, 24%; female = 28%, male = 19%), ankle tape (*n* = 33/401, 8%; female = 8%, male = 9%) and shock‐absorbing insoles (*n* = 21/401, 5%; female = 5%, male = 5%) (Figure 1). Ankle braces were less frequently used (<5%). Forty‐two reasons were provided in free text as to why participants used supplementary ankle tape or external ankle supports, with the most common reasons being due to previous or current injury (*n* = 31/42, 74%), increased support (*n* = 4/42, 10%), injury prevention (*n* = 3/42, 7%) and foot posture beliefs (*n* = 3/42, 7%). A less frequent reason that was cited was that they were prescribed by a health professional (*n* = 1/42, 2%). Seventeen free text responses were provided regarding whether the use of ankle tape or braces affected footwear selection, with the most common reasons being they had no influence (*n* = 12/17, 71%), it does influence footwear selection, but no reason was provided (*n* = 3/17, 18%), and footwear needs to accommodate bracing (*n* = 2/17, 12%). One hundred and eighteen free text responses were provided as to why participants used foot orthoses or shock‐absorbing insoles, with the most common reasons being due to previous or current injury (*n* = 48/118, 41%), foot posture beliefs (*n* = 45/118, 38%) and prescribed by a health professional (*n* = 9/118, 7%). Cushioning, injury prevention, support, comfort and increasing footwear durability were less common reasons cited (all <5%) for using foot orthoses or shock‐absorbing insoles. Free text responses (*n* = 53) were provided regarding whether the use of foot orthoses or shock‐absorbing insoles affected footwear selection, with approximately one‐third indicating they had no influence (*n* = 16/53, 30%). The remaining responses indicated that footwear selection is influenced as they need to accommodate foot orthoses (*n* = 17/53, 32%), it does influence footwear selection, but no reason was provided (*n* = 11/53, 21%), use of a specific footwear brand was stated, but with no reason provided (*n* = 4/53, 8%) and that footwear is required to have a removable insole (*n* = 3/53, 6%). Selecting footwear with a soft midsole and purchasing trustworthy brands were less commonly cited reasons (all <5%).

#### 3.2.3 Footwear advice and purchasing habits

Most participants (*n* = 362/401, 81%) indicated that they seek advice regarding the selection of field hockey footwear, with footwear/hockey store staff (*n* = 210, 52%), teammates (*n* = 188, 47%), podiatrists (*n* = 102, 25%), Internet (*n* = 95, 24%), physical therapists/physiotherapists (*n* = 76, 19%) and coaches (*n* = 41, 10%) being the most common sources (Figure 1). Sports physicians, personal trainers and ‘other sources’ of advice were less frequently used (all <5%). Most participants used their footwear for over 12‐months before replacing them with a new pair (>12 months, *n* = 211/401, 53%; 1012 months, *n* = 121/401, 30%; 7–9 months, *n* = 39/401, 10%; 3–6 months, *n* = 27/401, 7%) and most commonly paid A$150–199 for their footwear (A$150–199, *n* = 172/401, 43%; A$100–149, *n* = 119/401, 30%; A$200–249, *n* = 89/401, 22%). Participants rarely used their hockey footwear for less than three months before replacing them or spent less than A$99 or more than A$250 (all <5%; Figure 1).

### 3.3 Footwear preferences

#### 3.3.1 Footwear features

**FIGURE 2:**
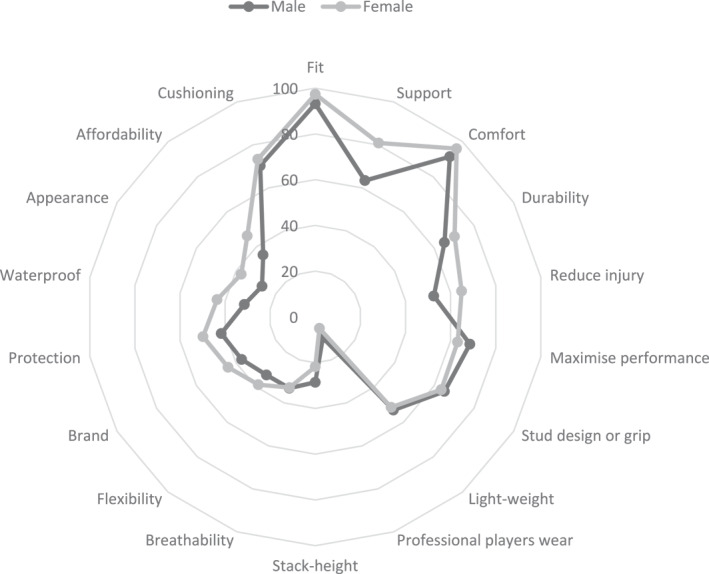
Percentage of participants rating a footwear factor as ‘important’ or ‘very important’ by sex (0%–100%). The non‐binary participant (*n* = 1) has not been included in the radar plot, as the small number of participants will under‐ or over‐state the percentage of frequencies for this gender.

**FIGURE 3:**
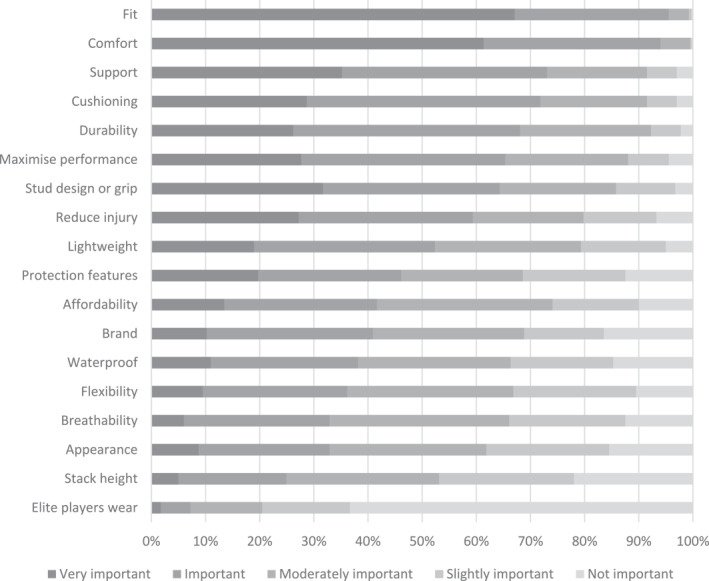
Importance of footwear features rated by field hockey players as percentage share (top to bottom = most important to least important, *n* = 401).

**FIGURE 4:**
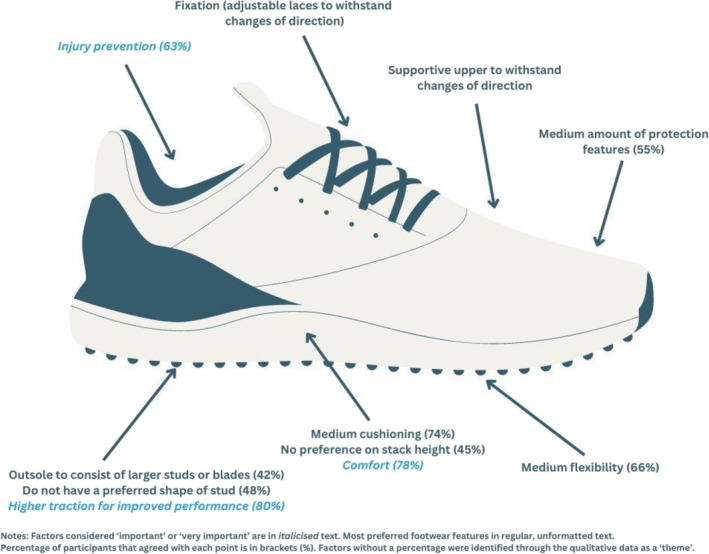
Important factors and most preferred features in footwear by field hockey players (*n* = 401).

When selecting field hockey footwear, fit (*n* = 269/401, 67%; female = 72%, male = 62%) and comfort (*n* = 246/401, 61%; female = 70%, male = 51%) were the only factors rated as ‘very important’ by most participants among 18 factors considered (Table 3) (Figure 2). The only factor that most participants rated as ‘not important’ was the footwear worn by elite players (*n* = 254/401, 63%; female = 65%, male = 61%) (Figure 3). When selecting hockey footwear outsole design (e.g., stud configuration, tread, etc.), most participants stated that higher traction for improved performance (*n* = 320/401, 80%), comfort (*n* = 313/401, 78%) and injury prevention (*n* = 253/401, 63%) were ‘important’ or ‘very important’ factors (Table 4). Participants indicated their preferred hockey outsole to consist of larger studs or blades, fewer in number and more spaced apart (*n* = 170/401, 42%) or many small studs or blades, closely grouped together (*n* = 138/401, 34%). One‐fifth of the participants indicated that they did not have a preference regarding outsole design (*n* = 80/401, 20%). Approximately half of the participants indicated that they did not have a preferred shape of stud (*n* = 193/401, 48%), with the remaining participants indicating they prefer a mix of different‐shaped studs (e.g., round, oval and bladed) (*n* = 93/401, 23%), bladed (*n* = 57/401, 14%) or round (*n* = 42/401, 11%). Few participants preferred no studs (e.g., road running footwear) or oval studs (all <5%) (Figure 4).

**TABLE 3:** Importance of factors when selecting footwear for field hockey (*n* = 401).

Factor	Not important	Slightly important	Moderately important	Important	Very important
Reducing injury risk	27 (6.7)	54 (13.5)	82 (20.4)	129 (32.2)	109 (27.2)
Maximise performance	18 (4.5)	30 (7.5)	91 (22.7)	151 (37.7)	111 (27.7)
Shoes elite players wear	254 (63.3)	65 (16.2)	53 (13.2)	22 (5.5)	7 (1.7)
Appearance	62 (15.5)	91 (22.7)	116 (28.9)	97 (24.2)	35 (8.7)
Brand	66 (16.5)	59 (14.7)	112 (27.9)	123 (30.7)	41 (10.2)
Breathability	50 (12.5)	86 (21.4)	133 (33.2)	108 (26.9)	24 (6.0)
Waterproof	59 (14.7)	79 (19.0)	113 (28.2)	109 (27.2)	44 (11.0)
Cushioning	12 (3.0)	22 (5.5)	79 (19.7)	173 (43.1)	115 (28.7)
Comfort	1 (0.2)	1 (0.2)	22 (5.5)	131 (32.7)	246 (61.3)
Fit	1 (0.2)	2 (0.5)	15 (3.7)	114 (28.4)	269 (67.1)
Durability	9 (2.2)	22 (5.5)	97 (24.2)	168 (41.9)	105 (26.2)
Affordability	40 (10.0)	64 (16.0)	130 (32.4)	113 (28.2)	54 (13.5)
Light‐weight	20 (5.0)	63 (15.7)	108 (26.9)	134 (33.4)	76 (19.0)
Stud design or grip	13 (3.2)	44 (11.0)	86 (21.4)	131 (32.7)	127 (31.7)
Stack height	88 (21.9)	100 (24.9)	113 (28.2)	80 (20.0)	20 (5.0)
Shoe flexibility	42 (10.5)	91 (22.7)	123 (30.7)	107 (26.7)	38 (9.5)
Support	12 (3.0)	22 (5.5)	74 (18.5)	152 (37.9)	141 (35.2)
Protection features	50 (12.5)	76 (19.0)	90 (22.4)	106 (26.4)	79 (19.7)

*Note*: Values are *n* (%).

**TABLE 4:** Importance of factors when selecting outsole designs for field hockey (*n* = 401).

Factor	Not important	Slightly important	Moderately important	Important	Very important
Comfort	13 (3.2)	6 (1.5)	69 (17.2)	162 (40.4)	151 (37.7)
Injury prevention	20 (5.0)	47 (11.7)	81 (20.2)	126 (31.4)	127 (31.7)
Higher traction/grip for improved performance	6 (1.5)	16 (4.0)	59 (14.7)	154 (38.4)	166 (41.4)

*Note*: Values are *n* (%).

When selecting hockey footwear, participants preferred medium cushioning (medium, *n* = 298/401, 74%; maximum, *n* = 70/401, 18%; no preference, *n* = 23/401, 6%), medium flexibility (medium, *n* = 263/401, 66%; no preference, *n* = 77/401, 19%; high, *n* = 35/401, 9%; low, *n* = 26/401, 7%) and medium amount of protection features (medium, *n* = 219/401, 55%; maximum, *n* = 71/401, 18%; no preference, *n* = 69/401, 17%; minimum, *n* = 42/401, 11%). Participants mostly had no preference for stack height (no preference, *n* = 179/401, 45%; medium, *n* = 165/401, 41%; low, *n* = 50/401, 13%). Few participants indicated a preference for minimum cushioning or a high stack height (all <2.5%). Twenty‐two free text responses were provided for ‘other’ factors considered when purchasing hockey footwear, with only two new themes identified, being fixation (e.g., adjustable laces) (*n* = 2) and a supportive upper able to withstand changes of direction (*n* = 2) (Figure 4).

### 3.4 Footwear beliefs and experiences

**FIGURE 5:**
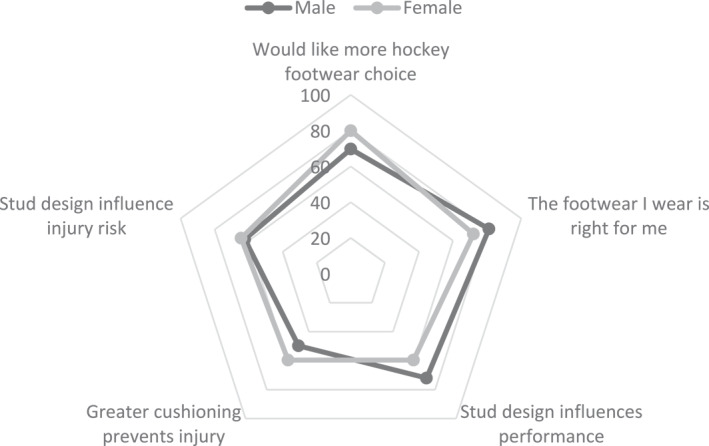
Percentage of participants rating a footwear factor as ‘agree’ or ‘strongly agree’ by gender (0%–100%). The non‐binary participant (*n* = 1) has not been included in the radar plot, as the small number of participants will under‐ or over‐state the percentage of frequencies for this gender.

Most participants ‘agree’ that the footwear they currently use for field hockey is right for them (*n* = 198/401, 49%; female = 44%, male = 57%), hockey stud design can greatly influence injury risk (*n* = 180/401, 45%, female = 44%, male = 46%), hockey footwear stud design can greatly influence athletic performance (*n* = 177/401, 44%; female = 42%, male = 47%) and greater cushioning in hockey footwear helps prevent injuries (*n* = 167/401, 42%, female = 45%, male = 37%). Most participants ‘strongly agree’ they would like more choice when purchasing hockey footwear (*n* = 164/401, 41%; female = 47%, male = 33%) (Table 5, Figure 5).

**TABLE 5:** Participant agreement with statements regarding field hockey footwear (*n* = 401).

Statement/belief	Strongly disagree	Disagree	Undecided	Agree	Strongly agree
Hockey footwear stud design can greatly influence my athletic performance (e.g. run faster, change direction quicker, etc.)	4 (1.0)	30 (7.5)	106 (26.4)	177 (44.1)	84 (20.9)
Hockey footwear stud design can greatly influence my injury risk	5 (1.2)	23 (5.7)	118 (29.4)	180 (44.9)	75 (18.7)
Greater cushioning in hockey footwear helps prevent injuries when playing hockey	9 (2.2)	44 (11.0)	127 (31.7)	167 (41.6)	54 (13.5)
The footwear that I am currently using for hockey games are right for me	6 (1.5)	29 (7.2)	62 (15.5)	198 (49.4)	106 (26.4)
I would like more choices when buying hockey footwear	4 (1.0)	35 (8.7)	59 (14.7)	139 (34.7)	164 (40.9)

*Note*: Values are *n* (%).

Eighty‐eight participants provided free text responses to the final survey question on any unaddressed factors, market gaps, or personal experiences related to hockey footwear selection, generating 145 coded items across 26 different themes (Supporting Information S3). The most common free text responses included a want for more fitting options (*n* = 25/88, 28%), especially for wide (*n* = 16/88, 18%) or narrow feet (*n* = 3/88, 3%) and for female's feet (*n* = 5/88, 6%). Participants also expressed challenges in obtaining suitable hockey footwear due to perceived inadequacy of current options (*n* = 20/88, 23%) or limited accessibility to product (*n* = 15/88, 17%). Additional concerns included insufficient foot protection (*n* = 10/88, 11%), a preference for trail running footwear for meeting player needs (*n* = 9/88, 10%), poor durability of current footwear (*n* = 9/88, 10%), the need for greater foot support (*n* = 6/88, 7%), comfort issues (*n* = 6/88, 7%) and the importance of adequate grip (*n* = 5/88, 6%). A further seventeen codes were identified from the responses (all <5 responses).

## 4 DISCUSSION

This study investigates the factors influencing field hockey players' footwear selection, perceptions regarding footwear design features on injury and performance, and experiences regarding usability. Our findings reveal that footwear attributes such as fit, comfort, support and cushioning are of most importance to hockey players, and there are sex‐dependent differences regarding footwear selection and fitting issues.

### 4.1 Footwear selection, usability and sex disparities

Most participants wore field hockey‐specific footwear (62%), but over one‐third used footwear designed for other sports, particularly trail running (34%). Notably, a higher percentage of female participants (40%) used trail running footwear compared to males (25%). Qualitative insights highlight the challenges females experience in sourcing appropriately fitted hockey‐specific footwear, most likely due to differences in foot width and foot height between males and females [16, 17]. This challenge is compounded by a lack of female‐specific options in the market. Most hockey footwear used by participants in this study were made from a 'male' or ‘unisex’ last with no width options, thereby favouring the shape of a male foot. Trail running footwear offers more fitting options, with some brands offering sex‐specific lasts and multiple widths, increasing the likelihood of finding appropriately fitted footwear and optimising footwear comfort [18]. While trail running shoes typically provide fewer protective features and less waterproofing compared to hockey‐specific footwear, our study revealed that participants did not prioritise these specific design elements. This, coupled with the greater fitting options, may help explain the popularity of trail running footwear among field hockey players.

A substantial proportion of participants (female = 47%; male = 33%) ‘strongly agree’ that they would like more choice when purchasing hockey footwear. This finding, along with the number of hockey players using trail running footwear, suggests potential usability issues with hockey‐specific footwear for many individuals. Despite the desire for more choice, approximately three‐quarters of participants expressed satisfaction with their current footwear, suggesting that a combination of hockey‐specific and trail running footwear may adequately meet the needs of most hockey players. However, with approximately one‐quarter of participants expressing uncertainty or dissatisfaction with their current footwear, there remains substantial room for improvement in meeting the needs of these players and providing greater choice for all.

Participants mainly sought advice on new hockey footwear from retailers and teammates, rather than from podiatrists, physiotherapists or sports physicians. These results are similar to those reported by Dhillon and colleagues [18], where runners preferred footwear advice from retailers and the Internet over healthcare professionals. While participants indicated wearing a wide variety of footwear brands, most of the usage, comprising over three‐quarters (76%), was attributed to three brands. Notably, one of these brands does not manufacture hockey‐specific footwear. Geographical differences in brand usage were also observed, with our data identifying varying popularity of certain brands across locations (see Table 2). For example, in Oceania, the most popular brand was worn by approximately half of the respondents, whereas in Europe, this same brand was only worn by one‐quarter of the respondents. Conversely, the most popular brand in Europe was also worn by half of the participants, while it was only worn by approximately 10% of respondents in Oceania. This observation suggests that brand usage may be influenced by various factors, including differing preferences of local players, brand recognition, market prominence and footwear accessibility.

### 4.2 Preferred footwear features and participant perceptions

When purchasing field hockey footwear, participants placed the most importance on footwear fit above all other factors, closely followed by comfort. As fit is a key factor associated with footwear comfort, it makes it a crucial consideration [19]. Additionally, participants also valued support and cushioning, with both known to affect overall footwear comfort [19]. Cushioning is not only valued by players for comfort, as most participants also expressed a perception that greater cushioning in hockey footwear can help prevent injuries. Previous studies evaluating the effect of footwear comfort in running [20] and football [21] have indicated that improved comfort can increase performance. In the absence of evidence‐based guidance on choosing running shoes to prevent injuries, it has been recommended that factors such as comfort, gradual transition to new shoes and listening to your body during training are valid considerations [22, 23]. In line with these principles, and given the limited research on field hockey footwear, it is logical that players continue to prioritise footwear comfort when selecting their footwear. Considering the complex and multifaceted nature of footwear comfort [19], further research into the influence of different hockey footwear features on comfort is warranted.

In our study, participants considered footwear‐surface traction (grip) as an important factor when selecting footwear. However, almost half of them had no preference for stud shape, and approximately one‐fifth showed indifference towards outsole design. Furthermore, no notable differences in stud preference were evident between playing positions, and participants did not alter their footwear based on playing surface. While most participants expressed a perception that stud design can influence athletic performance and injury risk, it is possible that they may not be aware of the relationship between outsole design, its interaction with the playing surface and its potential influence on performance and injury risk. Previous research has shown that different stud configurations in football boots result in varying magnitudes of rotational traction [24], and higher levels of rotational traction are associated with increased lower limb injury risk in American Football athletes [25]. Given this knowledge of outsole–surface interactions in football, further research is needed to assess the effect of outsole design on injury and performance across different surfaces in field hockey, including hockey‐specific and trail running footwear. Effective communication of these findings to players could improve their awareness and decision‐making when selecting footwear for field hockey.

### 4.3 Study considerations and limitations

The findings of this study need to be considered within the context of four key limitations. First, as data were collected via an online survey there is a risk of recall bias that is inherent in self‐reported study design. Second, participant's levels of knowledge of footwear features may limit their ability to provide accurate answers pertaining to actual footwear design features. Consequently, the findings from this study should be interpreted solely as perceptions, as their level of knowledge around footwear features remains unknown. Third, it is important to note that our survey was only available to English speakers, and most responses came from Australian hockey players (77%). Consequently, our sample may not be representative of hockey players worldwide or those who did not participate in our survey, so their responses may not fully reflect the perspectives of the broader hockey‐playing population. Lastly, the cross‐sectional design of our study means that the data presented are only representative of the period during which it was collected.

## 5 CONCLUSIONS

Findings of this survey demonstrate that most field hockey players wear hockey‐specific footwear when playing, but more than one‐third do not. Trail running footwear was the next most worn footwear style and was particularly favoured by female hockey players. Hockey players reported that footwear fit, comfort, cushioning and support were the most important factors they consider when purchasing footwear for hockey. Hockey players also indicated a desire for more footwear fitting options. Further studies should evaluate the influence of different footwear design features on comfort, injury risk, performance and usability.

## AUTHOR CONTRIBUTIONS

**Christopher R. Derry**: conceptualization; methodology; data curation (lead); resources; formal analysis (lead); project administration; writing – original draft (lead); writing – review and editing. **Hylton B. Menz**: conceptualization; methodology; data curation; resources; writing – review and editing. **Katrine Okholm Kryger**: conceptualization; methodology; data curation; writing – review and editing. **Athol Thomson**: conceptualization; methodology; data curation; writing – review and editing. **Caoimhe Hoey**: conceptualization; methodology; data curation; writing – review and editing. **Daniel R. Bonanno**: conceptualization; methodology; data curation; resources; formal analysis (lead); project administration; writing – original draft (lead); writing – review and editing.

## CONFLICT OF INTEREST STATEMENT

Katrine Okholm Kryger and Athol Thomson have previously received research funding from sportswear manufacturing companies. Athol Thomson is a consultant for a sports footwear company. All other authors declare that they have no competing interests.

## ETHICS STATEMENT

Approval was provided by the La Trobe University Human Ethics Committee (HEC21321).

## Supporting information

Supporting Information S1

Supporting Information S2

Supporting Information S3

## Data Availability

Data sharing is not applicable to this article as no new data were created or analyzed in this study.
