# Supplementary material for: Factors that influence field hockey footwear selection: An online survey
Source: J Foot Ankle Res. 2024 May 29;17(2):e12019. doi: 10.1002/jfa2.12019 (PMC11296714; doi:10.1002/jfa2.12019)
Supplement: Supplementary file 2 — Supporting Information S2 [file JFA2-17-e12019-s002.docx]

| **FACTORS THAT INFLUENCE FIELD HOCKEY FOOTWEAR SELECTION** |
| --- |
| Thank you for agreeing to participate in this short survey.  Please note: your responses collected in this survey will remain anonymous.  This survey has been designed to understand the factors that influence the selection of hockey footwear. The findings from this survey will inform future hockey footwear research and development. The study is conducted by a team of experienced footwear researchers: Dr Daniel Bonanno^1^, Mr Chris Derry^1^, Prof Hylton Menz^1^, Dr Athol Thomson^1,2^, Miss Caoimhe Hoey^3^, Dr Katrine Okholm Kryger^4^  1. Discipline of Podiatry, La Trobe University, Melbourne, Australia  2. Aspetar Orthopaedic & Sports Medicine Hospital, Doha, Qatar  3. Bon Secours Hospital Galway, Republic of Ireland  4. Faculty of Sport, Health and Applied Science, St Mary’s University, Twickenham, London, England  **SECTION A: IN THIS SECTION WE WANT TO LEARN A BIT ABOUT YOU**   1. **What is your age (years)**  \|  \| \| --- \|   **2. To which sex do you most identify (select all that apply)?**  Female  Male  Non-binary  Prefer not to answer  **3. What is your height (cms)?**   \|  \| \| --- \|   **4. What is your weight (kgs)?**   \|  \| \| --- \|   **5. What is your shoe size (US size)?**  Note: if you don’t know your US shoe size, you can use the shoe size converter here:  Women <https://www.asos.com/au/discover/size-charts/women/shoes/>  Men <https://www.asos.com/au/discover/size-charts/men/shoes/>   \|  \| \| --- \|     **6. How many years have you been playing hockey?**   \|  \| \| --- \|   **7. What is your nationality?**   \|  \| \| --- \|   **8. What is your ethnicity (select one option)?**  OCEANIAN  Australian Peoples  New Zealand Peoples  Melanesian and Papuan  Micronesian  Polynesian    NORTH-WEST EUROPEAN  British  Irish  Western European  Northern European    SOUTHERN AND EASTERN EUROPEAN  Southern European  South Eastern European  Eastern European    NORTH AFRICAN AND MIDDLE EASTERN  Arab  Jewish  Peoples of the Sudan  Other North African and Middle Eastern    SOUTH-EAST ASIAN  Mainland South-East Asian  Maritime South-East Asian    NORTH-EAST ASIAN  Chinese Asian  Other North-East Asian    SOUTHERN AND CENTRAL ASIAN  Southern Asian  Central Asian    PEOPLES OF THE AMERICAS  North American  South American  Central American  Caribbean Islander    SUB-SAHARAN AFRICAN  Central and West African  Southern and East African  **9. In what country do you currently play hockey?**   \|  \| \| --- \|   **10. How many hockey sessions (training and games) do you complete in a typical week (when in-season, and when not affected by COVID19 restrictions)?**   \|  \| \| --- \|   **11. What hockey position do you mostly play?**  Striker  Midfield  Defender  Goalkeeper  **12. What is the highest level of hockey that you play?**  Organised recreational (e.g., club grades, social)  First grade (highest grade within your immediate region)  National league (highest grade within your country)  International (you represent your country)  Other (please specify)   \|  \| \| --- \|   **13. What type of surface(s) do you mostly play hockey on (can choose multiple answers)?**  Water-based synthetic  Sand-based synthetic  Hybrid synthetic (natural grass with artificial reinforcing fibres)  Grass  Other (please specify)   \|  \| \| --- \|   **SECTION B: IN THIS SECTION WE WANT TO LEARN ABOUT YOUR FOOTWEAR PREFERENCES**  **14. Which of the following categories best describes the shoes you currently use for hockey?**  Specific hockey shoes  Trail running shoes  Road running shoes  Synthetic/astro turf football shoes  Other (please specify)   \|  \| \| --- \|   **15. What shoes are you currently using for hockey? (If you are wearing more than one pair, please state your favourite/preferred shoe)?**   \| Preferred shoe (Brand/Model): \| \| --- \|   **16. Do you wear a different pair of shoes depending on the surface you are playing on?**  No  Yes, please specify why you do this and how the shoes are different   \|  \| \| --- \|   **17. How much do you typically pay for a pair of hockey shoes?**  Note: the below answers are in Australian Dollars (A$). You can use the currency converter table below or use the online currency converter here: <https://www.xe.com/currencyconverter/convert/?Amount=1.00&From=AUD&To=USD>  Less than A$99  Between A$100-149  Between A$150-199  Between A$200-249  More than A$250   \| Australian $ \| US $ \| EURO \| GBP \| Canadian $ \| \| --- \| --- \| --- \| --- \| --- \| \| A$100 \| $72 USD \| 61€ \| 55£ \| $95 CAD \| \| A$150 \| $108 USD \| 91.5€ \| 82.5£ \| $142.5 CAD \| \| A$200 \| $144 USD \| 122€ \| 110£ \| $190 CAD \| \| A$250 \| $180 USD \| 152.5€ \| 137.5£ \| $237.5 \|   **18. How long do you typically use your hockey shoes before replacing them with a new pair?**  Less than 3 months  3-6 months  7-9 months  10-12 months  more than 12 months  **19. When I play hockey, I typically use (you can select multiple answers):**  Ankle tape  Ankle brace  Foot orthoses  Shock-absorbing insole  None of the above  (If options 1-4 are selected) Please specify why you use this and if it influences your footwear selection?   \|  \| \| --- \|   **20. When purchasing hockey footwear, I make my decisions based on:**   \|  \| **Not important** \| **Slightly important** \| **Moderately important** \| **Important** \| **Very important** \| \| --- \| --- \| --- \| --- \| --- \| --- \| \| - **Reducing the risk of injury** \| 1 \| 2 \| 3 \| 4 \| 5 \| \| - **Maximising athletic performance** \| 1 \| 2 \| 3 \| 4 \| 5 \| \| - **The shoes that elite player/s wear** \| 1 \| 2 \| 3 \| 4 \| 5 \| \| - **Appearance (e.g., colour)** \| 1 \| 2 \| 3 \| 4 \| 5 \| \| - **Brand** \| 1 \| 2 \| 3 \| 4 \| 5 \| \| - **Breathability** \| 1 \| 2 \| 3 \| 4 \| 5 \| \| - **Waterproof** \| 1 \| 2 \| 3 \| 4 \| 5 \| \| - **Cushioning** \| 1 \| 2 \| 3 \| 4 \| 5 \| \| - **Comfort** \| 1 \| 2 \| 3 \| 4 \| 5 \| \| - **Fit** \| 1 \| 2 \| 3 \| 4 \| 5 \| \| - **Durability** \| 1 \| 2 \| 3 \| 4 \| 5 \| \| - **Affordability** \| 1 \| 2 \| 3 \| 4 \| 5 \| \| - **Light-weight** \| 1 \| 2 \| 3 \| 4 \| 5 \| \| - **Stud design or grip** - **Stack height (height of footwear material under your foot)** \| 1  1 \| 2  2 \| 3  3 \| 4  4 \| 5  5 \| \| - **Shoe flexibility (bending and twisting the shoe)** \| 1 \| 2 \| 3 \| 4 \| 5 \| \| - **Support** \| 1 \| 2 \| 3 \| 4 \| 5 \| \| - **Protection features (e.g., reinforced upper, toe guard)** \| 1 \| 2 \| 3 \| 4 \| 5 \| \| - **Other: Please state………………** \| 1 \| 2 \| 3 \| 4 \| 5 \|   **21. My preferred hockey shoe outsole (i.e. stud configuration, tread, etc.) is:**  Many small studs or blades, closely grouped together (e.g. dimpled outsole, see image 1 below)  Larger studs or blades, fewer in number and more spaced apart (see image 2 below)  No studs or blades (e.g. road running shoe)  I don’t have a preference  Other (please specify)   \|  \| \| --- \|   **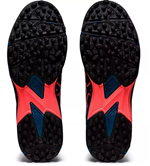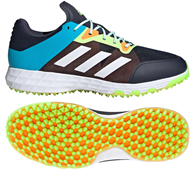**  **Image 1 (above):** Example of ‘many small studs or blades, closely grouped together’  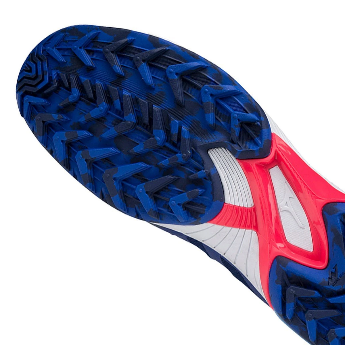 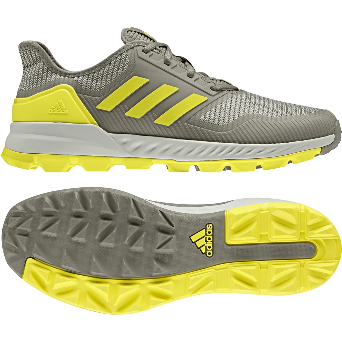  **Image 2 (above):** Example of ‘larger studs or blades, fewer in number and more spaced apart’  **22. I prefer the shape of my studs to be:**  Round  Oval  Bladed  A mixed of different shaped studs (e.g., round, oval and bladed)  I don’t have a preference  No studs (e.g., road running shoe)  Other (please specify)   \|  \| \| --- \|   **23. When selecting my hockey shoe outsole (i.e., stud configuration, tread, etc.), I make my decisions based on:**   \|  \| **Not important** \| **Slightly important** \| **Moderately important** \| **Important** \| **Very important** \| \| --- \| --- \| --- \| --- \| --- \| --- \| \| - **Comfort** \| 1 \| 2 \| 3 \| 4 \| 5 \| \| - **Higher traction/grip for improved performance (e.g., change in speed, change of direction, etc.)** \| 1 \| 2 \| 3 \| 4 \| 5 \| \| - **Injury prevention** \| 1 \| 2 \| 3 \| 4 \| 5 \|   **24. My preferred level of hockey shoe cushioning is:**  Minimum cushioning (i.e., hard/firm)  Medium cushioning (i.e., between soft and hard/firm)  Maximum cushioning (i.e., soft)  I don’t have a preference  **25. My preferred level of hockey shoe stack height (i.e., thickness of shoe under the heel, including outsole, midsole, and insole) is:**  Low stack height (13mm or less)  Medium stack height (14mm to 25mm)  High stack height (26mm or greater)  I don’t have a preference  **26. My preferred level of hockey shoe flexibility (bending and twisting) is:**  Low flexibility (i.e., hard to bend and/or twist the shoe)  Medium flexibility (i.e., moderate force needed to bend and/or twist the shoe)  High flexibility (i.e., easy to bend and/or twist the shoe)  I don’t have a preference  **27. My preferred hockey shoe has the following amount of “protection” features:**  Minimum protection features (no reinforcement in the shoe, no toe guard, etc)  Medium protection features (has reinforcement in 1 or 2 areas of the shoe, may have a toe guard)  Maximum protection features (several reinforced areas of the shoe exist, solid toe guard, etc.)  I don’t have a preference  **SECTION C: IN THIS SECTION WE WANT TO LEARN ABOUT YOUR BELIEFS AND EXPERIENCES REGARDING HOCKEY FOOTWEAR**  **28. Where do you seek hockey footwear advice from (select all that applies)?**  I would not seek advice  Shoe/hockey store salesperson  Podiatrist  Physical therapist/Physiotherapist  Sports physician  Personal trainer  Internet  Teammates  Coach  Other (please specify)   \|  \| \| --- \|   **29. Please read the statements below and rate how strongly you agree or disagree:**   \|  \| **Strongly disagree** \| **Disagree** \| **Undecided** \| **Agree** \| **Strongly agree** \| \| --- \| --- \| --- \| --- \| --- \| --- \| \| - **Hockey footwear stud design can greatly influence my athletic performance (e.g., run faster, change direction quicker, etc.)** \| 1 \| 2 \| 3 \| 4 \| 5 \| \| - **Hockey footwear stud design can greatly influence my injury risk** \| 1 \| 2 \| 3 \| 4 \| 5 \| \| - **Greater cushioning in hockey footwear helps prevent injuries when playing hockey** \| 1 \| 2 \| 3 \| 4 \| 5 \| \| - **The footwear that I am currently using for hockey games are right for me** \| 1 \| 2 \| 3 \| 4 \| 5 \| \| - **I would like more choice when buying hockey footwear** \| 1 \| 2 \| 3 \| 4 \| 5 \|   **30. Is there anything else you wish to tell us about factors that influence your selection, gaps in the market, or experiences (good or bad) regarding hockey footwear?**  No  Yes, please specify:   \|  \| \| --- \|   **THANK YOU FOR TAKING THE TIME TO COMPLETE THIS SURVEY!** |
