# Supplementary material for: Factors that influence field hockey footwear selection: An online survey
Source: J Foot Ankle Res. 2024 May 29;17(2):e12019. doi: 10.1002/jfa2.12019 (PMC11296714; doi:10.1002/jfa2.12019)
Supplement: Supplementary file 3 — Supporting Information S3 [file JFA2-17-e12019-s003.docx]

Additional file 2: Unaddressed factors, market gaps, or personal experiences related to hockey footwear selection

| **Gaps and issues when selecting hockey footwear** | | | | |
| --- | --- | --- | --- | --- |
| Topic | Sub-theme | | N | Example quote |
| Footwear fit issues | Need wider | | 16 | The biggest issue for me is finding shoes that fit both my orthotic and my wide foot……The biggest gap in the market is the wide and deep fitting hockey-specific shoe. |
|  |  | |  | Would like to see different widths available. |
|  |  | |  | There are a lot of bigger hockey players that would benefit from a decent pair of supportive comfortable shoes, wider anti ankle roll shoes |
|  |  | |  | Wish hockey shoes provided more room for toes to spread out and move when running. |
|  |  | |  | Width of shoe. Most brands……have a very narrow foot. I go without preferred extra safety things (like metal toe guards) for improvement in comfort of fit. |
|  |  | |  | I choose Mizuno wave ascends because they have a wider toe fitment suited to my foot shape. If it doesn't fit right I won't buy it regardless of how good they look or perform. |
|  |  | |  | Width is super important - I've ended up with metatarsalgia as a result of wearing shoes that were too narrow. |
|  |  | |  | I find a lot of hockey specific shoes have a narrow toe box, this doesn't allow for proper movement of the toes |
|  |  | |  | more variety in wide last shoes |
|  |  | |  | I would have happily spent more if the fit and comfort and feel around my heel and across the top of my foot were perfect. Most hockey shoes aren't made for wide feet (eee). |
|  |  | |  | Not enough wide foot options |
|  |  | |  | Very limited range and accessibility of extra wide trail shoes (let alone hockey specific - a dream at this stage) |
|  |  | |  | make some more options for wide feet. |
|  |  | |  | Women's shoes are not wide enough so I buy men's shoes. |
|  |  | |  | I purchase kids trail runners to accommodate my shorter wider feet (note: women participant) |
|  |  | |  | There aren't enough women shoes with a wide toe section. I have to go to the men’s shoes sometimes as the toes are to narrow |
|  | Shoe sizing issue | | 5 | Availability of sizing and women's shoes generally. I'm a half size which limits my shoe options greatly and makes it hard to find a good fit |
|  |  | |  | I only buy mens Brooks adrenaline shoes as a woman because ladies shoes don't go above a size 11 ladies. |
|  |  | |  | It would be amazing to be able to buy left and right shoe of the same brand with independent size. I am a 6 in my right foot and a size 5 in my left foot. |
|  |  | |  | There are not many shoes available in my size. |
|  |  | |  | There is not selection and between brands can variety of size different |
|  | Need narrower | | 3 | They tend to be very broad fitting. |
|  |  | |  | I have narrow feet, so getting a good fit for Hockey shoes is difficult as there is such a limited selection. In the past I have used trail running shoes instead. |
|  |  | |  | No options for people who have narrow feet. I have narrow feet and have to buy the standard size shoes which are often not a perfect fit |
|  | Preferred fit | | 1 | Shoe must fit firmly the forefoot and heel. No slop. |
| Inadequate options/unmet need |  | | 14 | The idea of a trail shoe for hockey is great (great grip, supportive shoe, water resistant), but I feel better toe protection is needed for hockey. |
|  |  | |  | It is very difficult to get a protection in a shoe that is also waterproof in a large size (>12UK) |
|  |  | |  | Finding good hockey shoes is really difficult. I find that most shoes are too heavy and are uncomfortable. |
|  |  | |  | Would like to see different widths available. |
|  |  | |  | I prefer a solid exterior to a shoe, opposed to mesh, but will not wear leather shoes for ethical reasons. At times this has limited my choice of appropriate footwear. |
|  |  | |  | Gap in the market for hockey shoes that have all the necessary features and look attractive. |
|  |  | |  | Hockey shoes have terrible cushioning at the moment. You have to go for a trail shoe which doesn't function like a hockey shoe. I feel like I have minimal options at the moment. A kayano with a trail bottom would be the ultimate for me. |
|  |  | |  | There is very little choice, particularly for women. Recommended shoes on footwear websites often group women's cricket shoes with hockey shoes but they're a completely different playing surface. |
|  |  | |  | There a quite a few gaps in the market for hockey shoes specific for hockey |
|  |  | |  | Market needs more options for a more comfortable hockey shoe e.g. trail shoe that doesn't compromise on protection |
|  |  | |  | The best shoes I've worn were designed for touch football, but they're no longer produced. |
|  |  | |  | Width of shoe…….I go without preferred extra safety things (like metal toe guards) for improvement in comfort of fit |
|  |  | |  | It would be amazing to be able to buy left and right shoe of the same brand with independent size. I am a 6 in my right foot and a size 5 in my left foot. |
|  |  | |  | Poor protection in the toe area. Looking at purchasing Lacrosse shoes as an option. |
|  | Goalkeeper specific needs | | 6 | 'Hockey shoes' typically have good studs and are hard all over which is great for toe protection, but are uncomfortably hard and typically have hard soles with little cushioning. They also seem to be too high around the ankle which is challenging with shin pads! Because you're spending at least 2 hours a session in the same shoes, I prefer comfort with cushioning. This is typically what trail shoes provide, as well as appropriate grip. However, typically, toe protection is minimal and seems to only be greater with gore-tex shoes that have no breathability!! I will keep searching! |
|  |  | |  | A shoe which has space for the kicker straps or which has spikes which can be unscrewed and removed like some running spikes would work well for goalkeepers |
|  |  | |  | There aren't many shoes that have a good grip pattern for Goalkeepers. The dimpling can cause our straps to move. Effecting play and the gear itself. |
|  |  | |  | There is a definite lack of footwear for goalkeepers. A lot of goalkeepers have to rely on cutting out studs to fit the kickers. |
|  |  | |  | As a goalkeeper, I prefer to use normal trainers so I can 'slide' when making a save. Grip or studs wouldn't be useful to me |
|  |  | |  | I've tried shoes with studs and no studs for goalkeeping - studs wear down the straps faster, but they keep the straps in place better which I prefer. |
| Accessibility (2) |  | | 15 | Poor selection available in Australia. Imported last pair from overseas. |
|  |  | |  | Lack of retailers, major chains don't carry hockey shoes |
|  |  | |  | In NZ we are just starting to get access brands like Osaka and inov8. |
|  |  | |  | In my area there are no shops to go in and try a decent range of hockey shoes, so we end up buying online |
|  |  | |  | Always run out of stock and sizes |
|  |  | |  | Not a large range to choose from |
|  |  | |  | There is not selection |
|  |  | |  | My size (UK4.5) aren't always available in all different models/makes. |
|  |  | |  | I have difficulty getting a choice of hockey footwear due to my larger foot size. At size 12 often only one or two options available. |
|  |  | |  | There are not a lot of dedicated hockey shops around…..depending on the time in the season when I'm buying, there may not be a lot of choices available in my convenient hockey shop in my size. |
|  |  | |  | Very little choice of hockey specific footwear even at hockey specific stores. |
|  |  | |  | A main factor for my hockey shoe selection is colour because my feet overheat….it can be difficult getting new shoes when the current season's colour selection of your favourite model shoes is a dark colour. |
|  |  | |  | Good variation in models provides better service to all wearers |
|  |  | |  | I've started looking for new ones (hockey shoes), not a lot of options in Tamworth. |
|  |  | |  | A D-width fitting in a hockey-specific shoe is very difficult to find in Australia. |
| Protection features |  | | 10 | I find that a lot of shoes compromise protection for enhanced breathability. |
|  |  | |  | A lot of hockey shoes are trying to be lighter but take away the toe guards and as a defender it is very important. |
|  |  | |  | I have lost big toenails on both feet from impact. Definitely want toe protection |
|  |  | |  | Poor protection in the toe area. |
|  |  | |  | Current shoes seem oriented to trail walking/running and have very little protection for feet and toes if hit by a ball or stick. |
|  |  | |  | There aren't many options available particularly light weight but protective. |
|  |  | |  | I like them to feel like a good running shoe with added support, protection & grip. |
|  |  | |  | 'Hockey shoes' typically have good studs and are hard all over which is great for toe protection, but are uncomfortably hard and typically have hard soles with little cushioning. This is typically what trail shoes provide, as well as appropriate grip. However, typically, toe protection is minimal and seems to only be greater with gore-tex shoes that have no breathability!! I will keep searching! |
|  |  | |  | Hockey shoes tend to be too stiff for running fast, trail shoes are better for performance and avoiding running injuries (as opposed to ball injuries which hockey shoes are better for) |
|  |  | |  | I prefer a solid exterior to a shoe, opposed to mesh, but will not wear leather shoes for ethical reasons. At times this has limited my choice of appropriate footwear. |
| Trail footwear better meets needs |  | | 9 | Very limited range and accessibility of extra wide trail shoes (let alone hockey specific - a dream at this stage) |
|  |  | |  | Have had lower limb injuries in the past. Prefer playing in runners but don't feel they have adequate grip. Had worn grass cat shoes as a junior and found them REALLY heavy, which turned me off specific hockey shoes for ages and I would play in trail runners. |
|  |  | |  | The idea of a trail shoe for hockey is great (great grip, supportive shoe, water resistant), but I feel better toe protection is needed for hockey. |
|  |  | |  | Hockey shoes have terrible cushioning at the moment. You have to go for a trail shoe which doesn't function like a hockey shoe. A kayano with a trail bottom would be the ultimate for me. |
|  |  | |  | I have narrow feet, so getting a good fit for Hockey shoes is difficult as there is such a limited selection. In the past I have used trail running shoes instead. |
|  |  | |  | A lot of hockey shoes are trying to be lighter but take away the toe guards and as a defender it is very important. I use trail shoes as they seem to have this feature. |
|  |  | |  | …goalkeepers need decent grip, but due to the strapping of the goalkeeping kicker, the tread needs to be flat in the areas the strap runs under the shoes…. I have never had hockey-specific shoes because of this - I opt for trail running shoes that have very short spikes. |
|  |  | |  | My shoe selection is based on picking the equivalent trail shoe to the runners I use, so there is limited change. |
|  |  | |  | Hockey shoes tend to be too stiff for running fast, trail shoes are better for performance and avoiding running injuries |
| Durability |  | | 9 | Ability to last in high intensity games |
|  |  | |  | Durability of many brands is terrible |
|  |  | |  | Often the studs will wear down quicker than I actually wear through the shoe, resulting in my wearing shoes that fit and are comfortable, but perhaps not performing the best because some studs are worn down |
|  |  | |  | Shoes that don’t deteriorate as quickly as they currently do! |
|  |  | |  | First year played 185 games plus training...very good. Bought another pair same model lasted a third as many games. Got them replaced with new model by retailer as they seemed faulty! Again sole, lace & grip failure....seems quality of product poor. |
|  |  | |  | Grip wear too quickly on sand dressed. |
|  |  | |  | More waterproof durable shoes. |
|  |  | |  | I've found my hockey shoes (trail running shoes) got holes where my little toe goes after not much use, from stopping abruptly in games. |
|  |  | |  | I reverted back to hockey specific shoes……the tread on the trail runners was too soft and would wear through too quick. |
| Support |  | | 6 | Prefer a shoe that is more like a supportive running shoe but with grip/trail shoe with better studs |
|  |  | |  | Arch support is not often considered |
|  |  | |  | Arch support |
|  |  | |  | I would wish for more ankle support and stability. |
|  |  | |  | A lot of bigger hockey players that would benefit from a decent pair of supportive comfortable shoes, wider anti ankle roll shoes as well is also something to keep in mind for some people, a lot of injuries happen due to rolling ankles so more support there would be great and help with confidence. |
|  |  | |  | Needs to be Comfortable, with so much running in hockey I like them to feel like a good running shoe with added support, protection & grip. |
| Comfort |  | | 6 | A main factor for my hockey shoe selection is colour because my feet overheat when exercising and, as many games are during the day, the heat of the Australian sun (even in winter) can make my feet uncomfortably hot |
|  |  | |  | Needs to be comfortable |
|  |  | |  | 'Hockey shoes' typically have good studs and are hard all over which is great for toe protection, but are uncomfortably hard and typically have hard soles with little cushioning… Because you're spending at least 2 hours a session in the same shoes, I prefer comfort with cushioning. This is typically what trail shoes provide, as well as appropriate grip. |
|  |  | |  | Ideally I would like a pair of hockey shoes that are the same fit and comfort as my trainers - but with more grip and protections… It's finding the right balance which give you the ability to change direction and no slipping over and adapting this to a comfortable shoe. |
|  |  | |  | I am prone to bad blisters due to my heel shape. A hard back with minimum cushioning is not suitable for me |
|  |  | |  | Finding good hockey shoes is really difficult. I find that most shoes are too heavy and are uncomfortable. |
| Grip |  | | 5 | I like them to feel like a good running shoe with added support, protection & grip. |
|  |  | |  | Grips wear too quickly on sand dressed. Grip isn't great on dry pitches I've found, almost friction like and feel more at risk of injury as can't trust the grip. |
|  |  | |  | More advice on what studs are most suitable per surface. It's finding the right balance which give you the ability to change direction and no slipping over and adapting this to a comfortable shoe. |
|  |  | |  | Prefer a shoe that is more like a supportive running shoe but with grip/trail shoe with better studs |
|  |  | |  | The material the studs are made out of - more plastic like or more rubbery - I feel the rubber gives better grip, the plastic studs lose grip well before the shoe itself wears out. |
| Weight |  | | 4 | There aren't many options available particularly light weight but protective. |
|  |  | |  | They tend to be very broad fitting and heavy. |
|  |  | |  | I find that most shoes are too heavy. |
|  |  | |  | The cheap shoes by hockey brands are usually too heavy and clunky. |
| Flexibility |  | | 3 | Hockey shoes tend to be too stiff for running fast, trail shoes are better for performance and avoiding running injuries (as opposed to ball injuries which hockey shoes are better for) |
|  |  | |  | Shoes can be too stiff, in relationship between sole and upper, ankle "socks" can be restrictive, grips wear too quickly on sand dressed. Grip isn't great on dry pitches I've found, almost friction like and feel more at risk of injury as can't trust the grip |
|  |  | |  | Wish hockey shoes provided more room for toes to spread out and move when running. Hockey shoes currently discourage healthy spread of feet during foot planting. Also hockey shoes tend to be very rigid, would prefer more flexibility. |
| Cushioning |  | | 3 | For me personally cushioning is important due to my stiff foot and personal preference, however in general I don't think greater cushioning will prevent injuries, I believe it is personal choice. |
|  |  | |  | I prefer a bit more cushioning and a medium stack to press off. |
|  |  | |  | 'Hockey shoes' typically have good studs and are hard all over which is great for toe protection, but are uncomfortably hard and typically have hard soles with little cushioning. |
| Lack of innovation |  | | 1 | Manufacturers bring out a 'new' shoe annually, its the old shoe but different colours. pointless. |
| Waterproof |  | | 2 | More waterproof durable shoes |
|  |  | |  | Ability to last in high intensity games and being wet for long periods and not retaining a bad smell when dry |
| Footwear advice |  | | 1 | More advice on what studs are most suitable per surface. It's finding the right balance which give you the ability to change direction and no slipping over and adapting this to a comfortable shoe. |
| **Experiences with hockey footwear selection** | | | | |
| Topic | | Sub-theme | N | Example quote |
| Cost | |  | 4 | Price and colour are huge |
|  | |  |  | Price is a large factor - as I'm only wearing the shoe approx twice a week, I don't like to spend a lot of money on them |
|  | |  |  | The price of genuine Hockey shoes in Australia is obscene. Between $250-$300 is poor value. |
|  | |  |  | Mostly the price is the deciding factor when I buy Hockey shoes! |
| Fit orthoses/brace | |  | 4 | The ability to easily connect extra ankle support equipment i.e Exo-ligament |
|  | |  |  | Many athletes now use orthotics and shoes that compliment this affects shoe choice. |
|  | |  |  | My shoe selection is based on picking the equivalent trail shoe to the runners I use…These also fit my orthotics |
|  | |  |  | The biggest issue for me is finding shoes that fit both my orthotic and my wide foot. |
| Purchasing habits | |  | 4 | I hate shoe shopping. So I found a brand and a size that fit, and therefore am very unlikely to switch, since I can now safely order online. |
|  | |  |  | Usually buy 3-4 pairs of the same pair of shoes to use over 3 years so I don't have change brand/style often. |
|  | |  |  | There are not a lot of dedicated hockey shops around, and unless I've had the exact same shoe before, I'm reluctant to buy online as I think it is important to try the shoe on for fit and comfort. Also I tend to only buy new shoes when old pair is either falling apart (used to be the primary problem, not so much these days) or when I notice that I'm starting to slip on the field (such as running out of Goal in Defence on penalty corner) as the tread has subtly worn down. |
|  | |  |  | In my area there are no shops to go in and try a decent range of hockey shoes, so we end up buying online |
| Aesthetics | |  | 4 | …need to improve their colours and design for female shoes |
|  | |  |  | Price and colour are huge in addition to function, good variation in models provides better service to all wearers, elite players opt for more low profile than other groups |
|  | |  |  | Gap in the market for hockey shoes that have all the necessary features and look attractive. |
|  | |  |  | A main factor for my hockey shoe selection is colour because...can make my feet uncomfortably hot before I even begin exercising if I am wearing dark (especially black) shoes. |
| Quality | |  | 2 | Several hockey brands have tried to get into the shoe market including Gryphon but they are never very good whereas ASICS are a shoe company so typically better. |
|  | |  |  | In NZ we are just starting to get access brands like Osaka and inov8. These smaller brands have great shoes. The cheap shoes by hockey brands are usually to heavy and clunky. |
| Evidence based claims | |  | 1 | Please conduct proper research before stating claims like 7.1 7.2 and 7.3. |
| Injury beliefs | |  | 1 | For me personally cushioning is important due to my stiff foot and personal preference, however in general I don't think greater cushioning will prevent injuries, I believe it is personal choice. |
| Selection habits | |  | 1 | My shoe selection is based on picking the equivalent trail shoe to the runners I use, so there is limited change. |
| Injury | |  | 1 | Have had lower limb injuries in the past. Prefer playing in runners but don't feel they have adequate grip. Had worn grass cat shoes as a junior and found them REALLY heavy, which turned me off specific hockey shoes for ages and I would play in trail runners. |
| Influences on shoe choice | |  | 1 | Word of mouth is also an influencing factor amongst Masters, who often experience age related injuries and have experienced recovery, rehab and repeat injuries. |
| Contracted player | |  | 1 | National and International representative players are likely to have endorsements with particular shoes companies - I myself as a National player have an endorsement and am limited within which models I am able to use. |
| Surface | |  | 1 | Differing surfaces (warm-ups on grass fields to then play on turf), age of synthetic turfs (difference in rebound forces) and the very limited range and accessibility of extra wide trail shoes (let alone hockey specific - a dream at this stage). |
